# GAS flaring and cancer in the Amazon of Ecuador: an ecological study

**DOI:** 10.1080/16549716.2026.2732789

**Published:** 2026-09-24

**Authors:** Miguel San Sebastian, Francesco Facchinelli, Wilmer Tarupi, Sara Castel-Feced

**Affiliations:** a Department of Epidemiology and Global Health, Umeå University, Umeå, Sweden; b Instituto de Epidemiología y Salud Comunitaria “Manuel Amunárriz”, Hospital Franklin Tello, Orellana, Ecuador; c Department of Historical and Geographic Sciences and the Ancient World, University of Padova, Padova, Italy; d National Cancer Registry – Sociedad de Lucha contra el Cáncer, SOLCA, Núcleo de Quito, Quito, Ecuador; e Department of Statistical Methods, Zaragoza University, Zaragoza, Spain

**Keywords:** Gas, oil, cancer, Amazon, Ecuador

## Abstract

**Background:**

Gas flaring associated with oil extraction is a major source of environmental pollution and has been linked to adverse health outcomes worldwide. In the Ecuadorian Amazon, decades of oil exploitation have resulted in widespread gas flaring, disproportionately affecting Indigenous and rural communities.

**Objective:**

This study assessed the association between exposure to gas flaring and cancer incidence in the provinces of Sucumbíos and Orellana, Ecuador, between 2010 and 2019.

**Methods:**

We conducted an ecological study at the parish level using data from the Quito Cancer Registry, which records diagnosed cancer cases among residents of Sucumbíos and Orellana. Exposure was defined according to the presence and number of active gas flaring sites identified through participatory mapping and spatial analysis. Age-standardised cancer incidence rates were calculated by sex and period (2010–2014; 2015–2019). Relative risks (RR) with 95% confidence intervals were estimated by comparing exposed and unexposed parishes.

**Results:**

A total of 1484 cancer cases were identified. Cancer incidence was higher in parishes exposed to gas flaring than in non-exposed parishes for both men and women in both periods. Relative risks ranged from 2.11 to 3.98 among men and from 1.53 to 3.28 among women, all statistically significant. Among women, a clear exposure–response gradient was observed, with higher cancer incidence in parishes, with more flaring sites.

**Conclusions:**

Parish-level exposure to gas flaring was strongly associated with higher cancer incidence in the Ecuadorian Amazon. These findings highlight the need for environmental mitigation, strengthened cancer surveillance, and targeted public health interventions in oil-affected regions.

## Paper Context

- **Main findings**: Cancer incidence was consistently higher in parishes exposed to gas flaring than in non-exposed parishes in the Ecuadorian Amazon, with a clear exposure–response pattern among women.
- **Added knowledge**: This study provides new evidence that gas flaring, as a specific source of pollution linked to oil extraction, is associated with increased cancer incidence in oil-affected communities.
- **Global health impact for policy and action**: The findings support urgent action to eliminate gas flaring, strengthen cancer surveillance, and improve prevention, early detection, and cancer care in environmentally exposed communities.

## Background

The burning of natural gas associated with oil extraction is a persistent practice and a growing concern internationally. In 2024, approximately 151 billion cubic metres (bcm) of gas were burned, the highest figure recorded since 2007 [1]. In terms of emissions, these practices released 389 million tonnes of CO_2_ equivalent (CO_2_e), including emissions from 46 million tonnes of unburned methane [1]; this amount is roughly equivalent to the greenhouse gas emissions of some 77 million cars. The scale of wasted energy is considerable; for example, the gas burned in 2024 could have supplied the annual gas consumption of the entire African continent [2].

This widespread gas burning has multiple consequences that affect both the environment and local communities. As mentioned above, it releases large amounts of greenhouse gases, mainly carbon dioxide and methane, which contribute significantly to global climate change [3–6]. However, its effects are not limited to the atmosphere: gas combustion also generates pollutants, such as nitrogen oxides (NO*_X_*, principally NO and NO_2_), sulphur dioxide (SO_2_), soot, fine particular matter and carcinogenic compounds, including benzene and polycyclic aromatic hydrocarbons (PAHs) [3–11]. These emissions provide a biologically plausible pathway linking long-term exposure to gas flaring with cancer development. Added to this is an economic component: the gas burned represents a valuable resource that could be used to generate electricity, which constitutes an especially unfair energy and financial loss in countries and regions where there is still a marked energy deficit [9,12–15].

Several studies have shown how people living near gas flaring sites are exposed to significant health risks. Prolonged exposure to air pollution has been associated with chronic diseases, while the constant heat, noise and presence of flames disrupt daily life and reduce the quality of life of local communities [16,17]. Oil and gas production in the United States has been estimated to be responsible for 7500 premature deaths, 410,000 asthma exacerbations and 2200 new cases of asthma in children each year [18]. Other US-based studies have shown that living within 5 km of gas flares can increase the risk of premature births [19] and that the total number of hospital visits for respiratory problems in people living within 60 mi of an active oil well would decrease by 33% (11,887 cases) if current environmental legislation were complied with [20].

Similarly, numerous studies from Nigeria have consistently shown an association between gas flaring from oil exploitation and an increase in respiratory and skin diseases and anaemia [21–23]. Furthermore, a review of the literature showed that oil spills and gas flaring can place pregnant women at high risk of hypertensive disorders of pregnancy, gestational diabetes mellitus, maternal depression and miscarriages [24].

Since the early 1970s, two provinces (Sucumbíos and Orellana) in the northeastern part of Ecuador have been affected by aggressive oil exploitation without adequate state control, leading to numerous environmental, social and health consequences for the local population [25–28].

According to World Bank estimates, gas flaring in Ecuador doubled between 2012 and 2024, rising from 800 to over 1.6 billion cubic metres per year [1]. A recent study that assessed the amount of gas flared and the removal of its pollutants in 53% of oil fields (*n* = 50/94) in the Amazon region from 2003 to 2012 estimated that 782 million cubic metres of gas were emitted annually, of which 470 t corresponded to black carbon, 6000 t of CO_2_ and 25 kt of methane [29]. Two more recent studies have provided more accurate details on the number of active flares in the Amazon region and on the population at risk, estimating that 99,918 people in Amazonian cities were in areas of moderate risk and 12,000 in areas of very high risk to their health due to direct exposure to flare emissions [30,31].

In response to this long history of environmental impacts, the ‘Eliminate Gas Flaring’ campaign was launched in 2018 by a network of national and international NGOs, Indigenous organisations, and civil society groups. The campaign aimed to end gas flaring in the region to protect Indigenous and rural communities living near flaring sites. As part of this process, nine Amazonian girls filed a legal protection action against the Ecuadorian state. On 29 July 2021, the Nueva Loja Court issued a landmark ruling banning gas flaring throughout the Ecuadorian Amazon, requiring oil companies to close flaring sites near populated areas within 18 months and eliminate all remaining sites by 2030 [32,33]. However, the full implementation of this ruling has yet to be realised.

While existing literature has examined the potential health impacts of oil exploitation in Ecuador, particularly in relation to cancer [34,35], no epidemiological studies have specifically investigated gas flaring as an independent exposure. The aim of this study was to analyse the relationship between exposure to pollutants emitted by gas flares and the cancer incidence in the provinces of Sucumbíos and Orellana, Ecuador, during the period 2010–2019.

## Methods

### Study population

Population-based cancer registries are considered the most reliable source of incidence and survival data, forming the basis for policy formulation and resource allocation in cancer care [36]. The Quito cancer register is a population-based registry whose primary catchment population comprises residents of Quito. In addition, the registry records cancer cases diagnosed in Quito among individuals residing outside the city. For the present study, we selected all cases registered between 2010 and 2019 among patients whose place of residence at the time of diagnosis was in the provinces of Sucumbíos or Orellana. The Registry’s sources of information are histopathological reports from pathology and haematology laboratories, as well as medical records from cancer care centres in the Comprehensive Public Health Network and Complementary Network [37].

This study utilized data on cancer patients diagnosed in the city of Quito who resided in the provinces of Sucumbíos and Orellana. Approximately 381,180 people live in these two provinces, according to the 2022 census [38].

Each province has a provincial hospital, but none offer histopathological services or access to cancer treatment. There are also no established cancer prevention programmes in these provinces or in the Amazon region.

### Cancer data

The data were provided by the National Cancer Registry of Quito. This registry contains detailed information on each patient, including a personal identification code, gender, age at diagnosis, cancer location, histology according to the International Classification of Diseases 10 (ICD-10), year of diagnosis, place of residence at the time of diagnosis. The inclusion of patient identifiers and diagnostic facility information facilitates case verification and prevents duplication, ensuring data quality [37]. For this study, all cases registered in the Quito registry corresponding to the provinces of Sucumbíos and Orellana from 2010 to 2019 were included.

As this was an anonymous registry-based study, patient involvement was not possible at any stage of the research process.

### Exposure to gas flaring

Data regarding the location of the gas flaring sites come from a study conducted by Facchinelli et al. [30]. Through a participatory process, local organisations and researchers mapped and documented 295 gas burning sites throughout the Amazon, of which 290 sites (432 flares) correspond to the provinces of Sucumbíos and Orellana. The authors compared the information obtained with several existing official datasets, including (a) annual data produced by the NOAA Nightfire algorithm for 2019; (b) daily detections from 1 January 2019 to 11 May 2020; and (c) official data from the Ecuadorian Ministry of the Environment. Given the long-term operation of oil fields in the region, with intensive oil activity and gas flaring occurring in the exposed parishes since the 1970s, flaring site locations were assumed to be relatively stable during the study period. However, historical information on the opening, closure, and activity levels of individual flaring sites was not available; therefore, temporal changes in individual flares could not be assessed.

In all cases, the data obtained recorded a higher number of gas flaring points than the other databases, constituting the most reliable open-access source on the distribution of gas flares in the Amazon. Spatial analyses were performed in QGIS v.3.34.14, comparing the location of the gas flaring sites with the parish map provided by the Military Geographic Institute (Figure 1). A map of the country with the location of the provinces is provided in Supplement 1.

**Figure 1.**
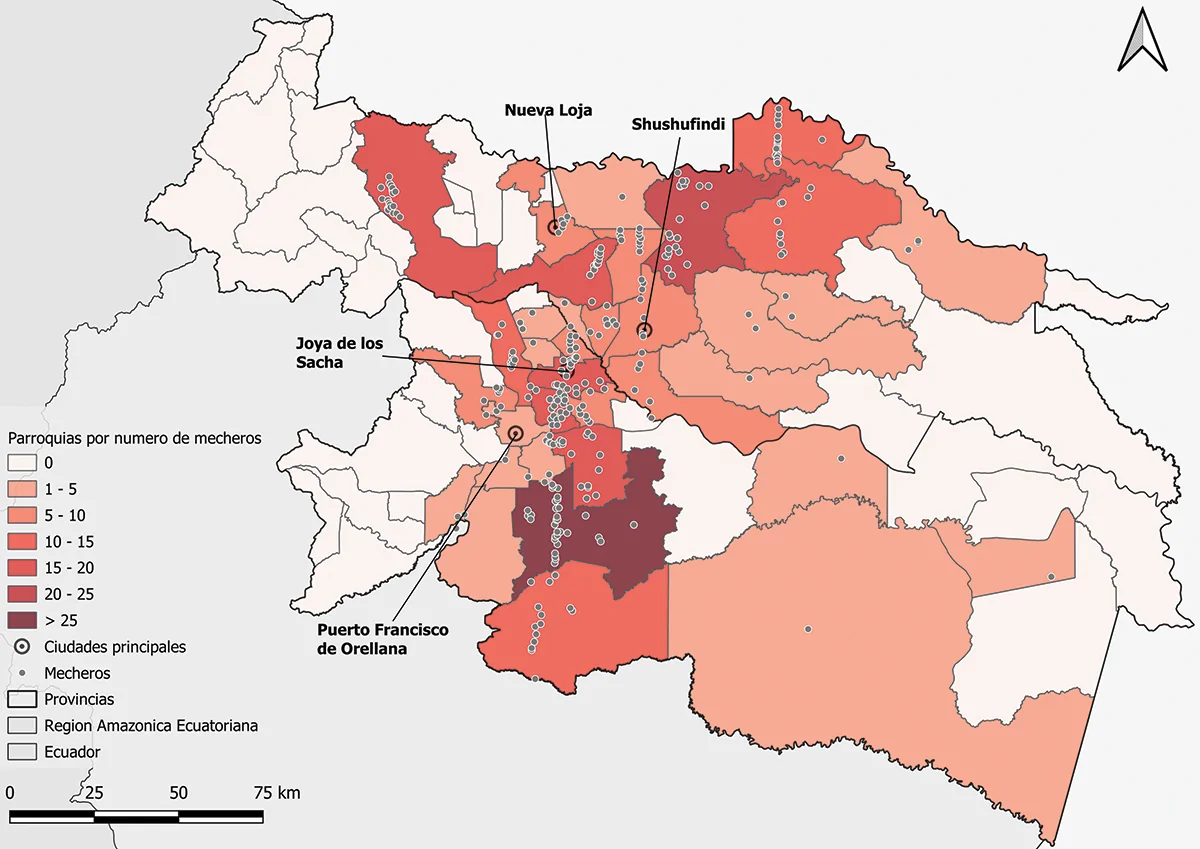
Location of gas flaring sites by parish in the provinces of Sucumbíos and Orellana.

The exposed population was defined as those living in parishes where there was at least one active gas flaring site. In total, 34 (52.3%) parishes out of 65 in the two provinces studied were considered exposed to gas flaring.

### Statistical analysis

First, the total number of cancer cases was calculated by sex, parish, and study period. Two periods were established to ensure enough cases: 2010–2014 and 2015–2019. Earlier periods were excluded due to administrative changes, including the creation of new parishes and name modifications, which made it difficult to accurately assign population data, cancer cases, and gas exposure sites to specific parishes.

Second, the incidence rates of all cancers combined were estimated according to exposure to gas flaring by sex. To calculate the denominator of the rates, population data from each parish were used, stratified by sex and 5-year age groups, choosing the population in the middle of the period as the denominator. These data were obtained from projections made by INEC based on the 2010 national census [39].

Third, age-standardised incidence rates for all cancers combined according to exposure to gas flaring and study period were calculated using the direct standardisation method and using Segi’s world population distribution [40] as the reference. The rates were divided by five (for the five years of each period) to obtain the average annual incidence. Finally, the relative risks (RR) and their 95% confidence intervals (95% CI) for men and women were estimated as ratios of age-adjusted incidence rates in parishes exposed and unexposed to gas flares.

These last two analyses were also complemented by another classification of exposure, dividing the exposed parishes into two groups: those with 1–5 gas flaring sites and those with more than five gas flaring sites.

The analyses were performed using the free statistical programme R.

## Results

### Number of cancer cases

A total of 1484 cases (35.2% in men) of cancer were recorded during the period 2010–2019 in the two provinces. An increase in diagnosed cases was observed in each period, both in men (around 25%) and women (around 22%). The cases of cancer distributed by exposure of parishes to gas flaring sites, by period and sex, are presented in Table 1.

**Table 1.** Distribution of cancer cases according to exposure to gas flaring, by period and sex (number and %).

	2010–2014		2015–2019		Total
Parishes	Men	Women	Men	Women	
Exposed	209 (90.1)	391 (90.5)	273 (93.8)	491 (92.8)	**1364**
Non-exposed	23 (9.9)	41 (9.5)	18 (6.2)	38 (7.2)	**120**
Total	232	432	291	529	**1484**

### Relationship between gas flaring and cancer

Figure 2a shows the age-standardised incidence rates in parishes exposed and not exposed to gas flaring, by sex. In both men and women, a higher incidence of cancer was observed in both periods in the group of parishes exposed to gas flaring.

**Figure 2.**
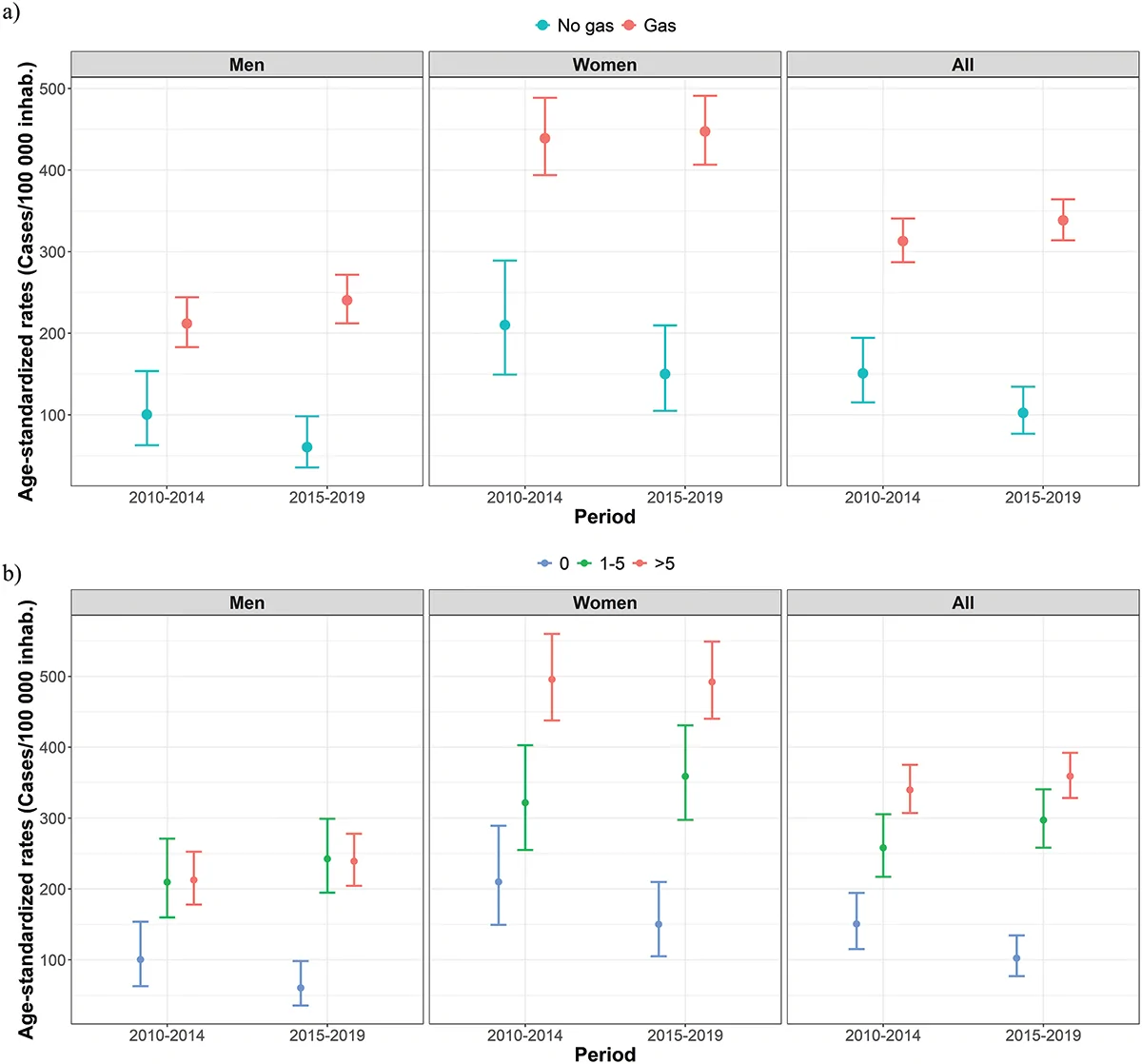
Age-standardised cancer incidence rates by sex, gas flaring exposure and study period. (a) Comparison between parishes with and without gas flaring sites. (b) Comparison across three exposure categories: no gas flaring sites, 1–5 sites, and >5 sites. Error bars represent 95% confidence intervals. Error bars represent 95% confidence intervals.

The rate ratios of these relationships are presented in Table 2. In men, the incidence of cancer was 111% higher in the 2010–2014 period and 298% higher in the 2015–2019 period in parishes with gas flaring. Similarly, for women, the incidence was 109% higher in the 2010–2014 period and 198% higher in the 2015–2019 period in parishes exposed to flares, compared to those not exposed. All these differences were statistically significant.

**Table 2.** Age-standardized incidence rates and rate ratios (RR) (in parentheses, their 95% confidence intervals) between parishes exposed and not exposed to gas flaring sites by period and disaggregated by sex.

		Men		Women
2010–2014	Rates*	RR (95% CI)	Rates*	RR (95% CI)
No flares Gas flares	100.3 211.8	1 2.11 (1.67–2.55)	210.0 439.0	1 2.09 (1.76–2.42)
2015–2019				
No flares Gas flares	60.5 240.5	1 3.98 (3.49–4.46)	150.0 447.2	1 2.98 (2.64–3.32)

*Age-standardized incidence rates ×100,000 inhabitants.

Figure 2b shows the age-standardised incidence rates by sex in unexposed parishes and in parishes exposed to gas flaring, further classified into two exposure levels. Cancer incidence rates were higher in exposed parishes at both exposure levels compared with unexposed parishes. Among men, incidence rates were similar across the two exposure categories. In contrast, among women, incidence increased progressively with a higher number of gas flaring sites in the parish.

The ratio of rates between parishes exposed and unexposed to different levels of gas sites, disaggregated by sex, are presented in Table 3. In men, the incidence of cancer was approximately 100% higher at both levels of exposure during the period 2010–2014 and 300% higher in the period 2015–2019 in parishes with gas flaring. In women, a gradual increase in incidence was observed as flares increased. In the period 2010–2014, the incidence was 53% higher in parishes with 1–5 flares and 136% higher in parishes with more than five flares. In the period 2015–2019, the incidences increased to 139% in parishes with 1–5 flares and 228% in those with more than five flares, compared to unexposed parishes. All differences were also statistically significant.

**Table 3.** Age-standardized incidence rates and rate ratios (in parentheses, their 95% confidence intervals between parishes exposed (two levels of exposure) and not exposed to gas flaring by period and disaggregated by sex.

	Men		Women	
2010–2014	Rates*	RR (95% CI)	Rates*	RR (95% CI)
No flares	100.3	1	210.0	1
1–5 flares	209.7	2.09 (1.60–2.58)	321.7	1.53 (1.15–1.91)
>5 flares	212.6	2.12 (1.67–2.57)	495.7	2.36 (2.02–2.70)
2015–2019				
No flares	60.5	1	150.0	1
1–5 flares	242.5	4.01 (3.50–4.52)	358.9	2.39 (2.02–2.77)
>5 flares	239.0	3.95 (3.46–4.44)	492.1	3.28 (2.94–3.62)

*Age-standardized incidence rates ×100,000 inhabitants.

## Discussion

This study demonstrates that the cancer incidence in the Amazonian provinces of Sucumbíos and Orellana was significantly higher in parishes exposed to gas flaring pollution than in non-exposed parishes. Relative risks were consistently elevated among both men and women, with a more pronounced dose–response relationship observed in women, where a higher number of flares were associated with a progressive increase in cancer risk.

While the literature linking air pollution and cancer is extensive [41,42], and numerous studies have documented various health impacts of gas flaring on nearby populations [18–24], to our knowledge, this is the first study to clearly document an association between exposure to gas flaring and an increased incidence of cancer. Previous studies have reported associations between oil and gas development, particularly unconventional oil and gas development, and cancer, but these studies did not specifically examine gas flaring as an exposure source [43,44].

The findings of this study are consistent with existing environmental and toxicological literature [45], which has shown that the incomplete combustion associated with gas flaring generates carcinogenic compounds such as polycyclic aromatic hydrocarbons, benzene, and fine particulate matter [3–6]. Epidemiological evidence linking exposure to these pollutants, particularly PAHs, with several cancers provides biological plausibility for the observed association. However, these pollutants are not specific to gas flaring, and this mechanistic evidence cannot distinguish the contribution of flaring from that of other environmental exposures associated with petroleum extraction.

## Methodological considerations

This study presents both strengths and limitations that should be acknowledged. One of its strengths is the use of data from the Quito National Cancer Registry, which is internationally recognized for its comprehensive coverage and rigorous data validation processes, thereby enhancing the reliability of the cancer incidence estimates. The analytical strategy also included calculating age-standardized rates and comparing exposed and non-exposed parishes, which helps reduce bias related to population structure.

As with any epidemiological study, however, there are methodological aspects that warrant careful consideration. The absence of a cancer registry in the Amazon region may lead to differential underreporting, as some patients may not have travelled to Quito for diagnosis. This could disproportionately affect remote areas or those with limited access to transportation. If access to diagnostic services differed systematically between parishes with and without gas flaring, differential under-ascertainment could have influenced the observed associations in either direction. As information on access to diagnostic services by parish was unavailable, the direction and magnitude of this potential bias could not be determined.

Furthermore, as this is an ecological study based on group-level characteristics and exposures rather than individual data, the findings should be interpreted with caution. Although an individual-level design would have been preferable, the use of smaller geographic units (parishes), rather than the cantons used in previous studies [46,47], helps to reduce some of the inherent bias in ecological analyses.

Individual-level information on other cancer risk factors (such as tobacco use, diet, or family history) was unavailable. Therefore, residual confounding by these or other lifestyle-related factors cannot be excluded. However, it is unlikely that these factors would be distributed differently based solely on the proximity to gas flaring sites.

Cancer incidence rates were calculated based on the parish of residence at the time of diagnosis, but information on duration of residence or previous places of residence was unavailable. Consequently, some individuals may have been classified as exposed or unexposed based on their current residence despite having had a different exposure history during the relevant period. This may have resulted in exposure misclassification. As information on migration patterns and duration of residence was unavailable, the direction and magnitude of this potential bias could not be determined.

Potential errors in population estimates should also be considered, as these could introduce bias into risk calculations. For instance, if exposed parishes experienced greater population growth than the non-exposed ones, this could lead to an underestimation of the incidence rates due to inflated denominators. However, population projections from the National Institute of Statistics and Censuses suggest that this has not occurred.

Moreover, the lack of detailed data prevented a longitudinal assessment of exposure. While existing documentation confirms environmental contamination from gas flaring in exposed parishes [28–30], the relevant exposure period for cancer development may date back one or two decades. Unfortunately, historical data on the location and volume of gas flared by different companies is not publicly available, making more precise analyses difficult. Nonetheless, all parishes classified as exposed have experienced intensive oil activity, and thus gas flaring, since the early 1970s [48,49]. Some exposure misclassification is however possible, particularly for the classification based on the number of flaring sites. Such misclassification should be considered when interpreting the observed associations and exposure–response patterns.

Another possible explanation for the elevated incidence of cancer near industrial sources is occupational rather than environmental exposure. However, although individual occupational data were unavailable, the fact that women in exposed parishes showed similar or even higher incidence than men reasonably argues against occupational exposure as the main explanation for the observed associations. The absence of a similar exposure–response pattern among men is more difficult to explain with the available data. Differences in work-related mobility could potentially reduce the accuracy of exposure classification based on parish of residence, without necessarily implying occupational exposure as the source of risk. However, information on mobility and occupational patterns was unavailable, and their contribution to the observed sex differences could not be assessed.

Finally, gas flaring occurs within a broader context of oil extraction, and parishes affected by flaring may also be exposed to other environmental hazards associated with petroleum operations. These include contaminants resulting from oil spills and the discharge of formation waters, which have been extensively documented in the region [29,50]. Other contextual differences between exposed and unexposed parishes may also contribute to the observed differences in cancer incidence. As information on these environmental and contextual factors was not available at the parish level, their potential influence could not be accounted for in the analysis. Consequently, although the study identified an association between parish-level gas flaring exposure and cancer incidence, the contribution of gas flaring cannot be fully separated from that of other environmental and contextual factors associated with oil extraction.

## Conclusions

A strong and statistically significant association was observed between parish-level exposure to gas flaring and cancer incidence at the parish level in the Ecuadorian Amazon. The consistency of the findings, the magnitude of the observed associations, the exposure-response pattern among women, and existing evidence on plausible biological mechanisms support the relevance of these results. They underscore the urgent need for targeted and effective environmental mitigation strategies to reduce community-level exposure to gas flaring, the establishment of a continuous cancer surveillance system in the region, and the development of an Amazonian oncology center equipped to provide prevention programs, early detection, accurate diagnosis, and comprehensive cancer care, including surgery and radiotherapy.

## Supplementary Material

Map_Ecuador_Supplement 1.tif

## Data Availability

The data for this study are available under request to the National Cancer Registry of Quito.
